# 离子液体功能化磁性Fe_3_O_4_纳米材料在样品前处理-色谱分析中的研究进展

**DOI:** 10.3724/SP.J.1123.2025.04025

**Published:** 2025-12-08

**Authors:** Jiaxin LI, Lizhu ZHAO, Xiangming SUN, Zhiqiang HE, Huiling CAO, Yingjin LUO, Bo YANG

**Affiliations:** 1.哈尔滨商业大学药学院，黑龙江 哈尔滨 150076; 1. College of Pharmacy，Harbin University of Commerce，Harbin 150076，China; 2.黑龙江省预防与治疗老年性疾病药物研究重点实验室，黑龙江 哈尔滨 150076; 2. Heilongjiang Key Laboratory of Preventive and Therapeutic Drug Research of Senile Diseases，Harbin 150076，China; 3.东北林业大学化学化工与资源利用学院，黑龙江 哈尔滨 150040; 3. College of Chemistry，Chemical Engineering and Resource Utilization，Northeast Forestry University，Harbin 150040，China

**Keywords:** 离子液体, 纳米材料, 固相萃取, 合成方法, 样品前处理, ionic liquids （ILs）, nanomaterials, solid-phase extraction （SPE）, synthesis methods, sample pretreatment

## Abstract

近年来，离子液体功能化磁性Fe_3_O_4_纳米材料（IL-Fe_3_O_4_ NPs）因稳定性好、吸附容量高、活性位点多、对有机或无机化合物的高溶解能力、可循环利用及易于分离等特点，广泛应用于样品前处理领域。离子液体具有结构可设计、导电性好、溶解能力强等特性，可单独或与其他材料共同用于修饰磁性Fe_3_O_4_纳米颗粒。这种修饰通过表面功能化，不仅能有效抑制纳米颗粒的团聚和氧化等缺陷，还能克服离子液体自身黏度高、传质效率低及分离困难等局限性，尤其适用于金属离子等痕量目标分析物的富集检测。目前，IL-Fe_3_O_4_ NPs已广泛应用于磁性固相萃取、管内固相微萃取及移液吸头固相萃取等前处理技术，并可与色谱、光谱等检测技术实现在线或离线联用，显著提升了检测的灵敏度与准确性，在食品安全、环境监测、生物医药等方面展现出巨大的潜力和发展空间。本文系统总结了IL-Fe_3_O_4_ NPs的合成方法、分类、萃取模式、在线或离线检测技术及在样品前处理的应用，并对该类材料未来可能的探索方向进行了展望。

## Abstract (en)

The analysis of complex sample matrices， such as environmental samples， food commodities， and biological specimens， requires sophisticated pretreatment methods. These techniques are fundamentally critical for isolating and enriching analytes of interest， thereby substantially enhancing the sensitivity， accuracy， and efficiency of subsequent analytical procedures. The judicious selection of adsorbent materials represents the pivotal element in achieving effective pretreatment. In recent years， ionic liquid-functionalized magnetic Fe_3_O_4_ nanoparticles （IL-Fe_3_O_4_ NPs） have garnered significant attention as highly promising materials within this domain. Their potential arises from an exceptional combination of properties： outstanding chemical and colloidal stability， high adsorption capacity， abundant surface active sites， superior solvation capabilities for diverse organic and inorganic compounds， potential for regeneration and reuse， and facile magnetic separation facilitated by an external magnetic field. Ionic liquids （ILs） are characterized by their structurally tailorable nature， excellent ionic conductivity， and potent dissolution capabilities. These intrinsic attributes render ILs highly effective as modifiers for Fe_3_O_4_ nanoparticles， either applied singularly or in hybrid composites with other functional materials. This surface functionalization fulfills two essential roles： firstly， it effectively mitigates the inherent tendency of nanoparticles towards agglomeration and provides a protective layer against oxidation； secondly， it circumvents well-documented limitations associated with bulk ionic liquids， notably their high viscosity （which impedes diffusion kinetics and mass transfer efficiency） and the practical difficulties often encountered in their separation from liquid phases. Consequently， IL-Fe_3_O_4_ NPs demonstrate particular utility for the efficient enrichment of trace-level analytes， such as metal ions. The resulting composite material successfully retains the advantageous core properties of the magnetic Fe_3_O_4_ substrate， specifically its superparamagnetic behavior （enabling rapid and efficient magnetic separation） and inherent biocompatibility. Simultaneously， it incorporates the highly desirable characteristics of ionic liquids， namely their extensive structural design flexibility and ease of chemical functionalization. The adsorption and extraction of analytes by IL-Fe_3_O_4_ NPs are governed by a complex interplay of multiple intermolecular forces. These encompass *π*-*π* stacking interactions， electrostatic attractions， hydrogen bonding， hydrophobic effects， and potentially coordinative interactions. This multifaceted binding capability underpins the material’s demonstrated high adsorption efficiency and selectivity towards various analytes. Currently， IL-Fe_3_O_4_ NPs are extensively employed across a broad range of modern sample pretreatment techniques. Principal methodologies include magnetic solid-phase extraction （MSPE）， in-tube solid-phase microextraction （IT-SPME）， and pipette-tip solid-phase extraction （PT-SPE）. Furthermore， these functionalized nanoparticles exhibit excellent compatibility for integration—both in online and offline configurations—with established analytical detection platforms. This includes coupling with chromatographic techniques such as high performance liquid chromatography （HPLC） and gas chromatography （GC）， as well as spectroscopic methods including atomic absorption spectroscopy （AAS） and inductively coupled plasma mass spectrometry （ICP-MS）. The seamless coupling of IL-Fe_3_O_4_ NPs-based extraction with these detection systems significantly augments overall method sensitivity and analytical accuracy. As a result， these materials show considerable promise for impactful applications in critical areas such as food safety assurance， environmental contaminant monitoring， and biomedical analysis. This article systematically summarizes the synthesis methods， classifications， main extraction modes， online or offline detection techniques， and applications in sample pretreatment of IL-Fe_3_O_4_ NPs， while also providing an outlook on potential future exploration directions for this class of materials.

随着样品基质越来越复杂、目标分析物浓度越来越低，开发兼具高效富集能力、优异选择性和良好稳定性的吸附材料已成为分析化学领域的研究热点之一，其材料的性能直接决定了目标化合物的选择性、富集效率以及方法的绿色化程度^［1］^。传统固相萃取材料常因比表面积有限、作用位点单一或化学稳定性不足，难以高效捕获复杂基质中的痕量目标物^［2］^。针对上述问题，亟须构建具备高比表面积、多功能活性位点及高稳定性的功能化材料，以满足现代分析化学对样品前处理的需求^［3］^。

磁性纳米颗粒（magnetic nanoparticles，MNPs）以其独特的纳米尺寸和高磁性已逐渐成为样品前处理的热点材料之一，主要包括Fe、Co、Ni等过渡金属氧化物及其复合物^［4］^。其中，磁性Fe_3_O_4_纳米材料（Fe_3_O_4_ NPs）因制备简便、成本低、易功能化修饰、粒径可控及生物相容性好等优势备受关注^［5］^。然而，Fe_3_O_4_ NPs在实际应用中面临易团聚、易氧化和选择性不足等挑战。为此，通常使用单层或多层无机或有机材料来包覆Fe_3_O_4_ NPs表面并形成功能性MNPs^［6］^。目前，常见的改性材料包括二氧化硅（SiO_2_）、碳基材料（如石墨烯、氧化石墨烯、碳纳米管和多壁碳纳米管）及金属有机骨架等材料^［7-10］^。而离子液体（ionic liquids， ILs）凭借出色的导电性、热稳定性、阴阳离子的可设计性和对有机金属化合物的强溶解能力，不仅能直接功能化Fe_3_O_4_ NPs表面，还可与上述改性材料协同修饰Fe_3_O_4_ NPs，在MNPs功能化中发挥着重要作用^［11，12］^。IL-Fe_3_O_4_ NPs通过*π*-*π*堆积、静电作用、氢键及疏水作用等机制，可高效富集多种目标化合物^［13］^。而且该材料在完成萃取过程后，无需离心操作，仅需施加外加磁场即可实现目标物质与样品的快速分离，具有萃取选择性强、操作简便且可重复利用等优势^［14］^。

近年来，已有文献报道了IL-Fe_3_O_4_ NPs的合成及应用。刘勤等^［15］^在2015年报道了IL-Fe_3_O_4_ NPs在磁性固相萃取（MSPE）中的应用，Chen等^［13］^则从表面功能化修饰的角度，系统总结了各种类型的IL-Fe_3_O_4_ NPs。然而，IL-Fe_3_O_4_ NPs在样品前处理领域仍在快速发展：一方面，新型离子液体（如膦酸酯功能化ILs、磺酸功能化ILs和具有双电荷结构的1，4-二氮杂双环［2.2.2］辛烷基ILs）不断被应用于合成新型功能化磁性纳米材料^［16-18］^；另一方面，MSPE、管内固相微萃取（IT-SPME）及移液吸头固相萃取（PT-SPE）等前处理方法也在不断完善，新的在线或离线技术不断涌现^［19-21］^。在此背景下，本文全面综述了IL-Fe_3_O_4_ NPs的合成方法、分类、萃取模式、主要检测技术及在样品前处理的应用，旨在为未来ILs在磁性纳米材料功能化方法及IL-Fe_3_O_4_ NPs的应用提出新的借鉴。

## 1 离子液体功能化磁性Fe_3_O_4_纳米材料的合成方法

IL-Fe_3_O_4_ NPs的合成主要包括两个关键步骤，首先是Fe_3_O_4_ NPs的合成，随后是ILs的进一步功能化修饰。

### 1.1 磁性Fe_3_O_4_纳米颗粒的合成

Fe_3_O_4_ NPs的合成主要包括3种典型工艺，分别为化学共沉淀法、水热/溶剂热法及溶胶-凝胶法^［22-24］^。化学共沉淀法作为制备Fe_3_O_4_ NPs的经典方法，主要通过将特定比例的Fe^2+^和Fe^3+^盐溶液与碱性沉淀剂（如氨水、NaOH）混合，在碱性条件下共沉淀生成Fe_3_O_4_ NPs^［25］^。该方法操作简便，适合大规模生产，但产物易团聚或氧化。水热/溶剂热法则以水或有机溶剂为介质，在高温高压密闭环境中促使铁盐前驱体分解结晶，可制得形貌规则、分散性良好的Fe_3_O_4_ NPs，但存在反应条件复杂、设备要求高、反应周期长等问题^［26］^。而溶胶-凝胶法以铁盐为原料，通过溶胶形成、凝胶化及热处理等步骤制备Fe_3_O_4_ NPs，具有产物纯度高、粒径分布窄且形貌可控的优势，但工艺步骤烦琐、耗时较长^［27］^。上述方法中，化学沉淀法凭借简便高效的特点，在Fe_3_O_4_ NPs的合成过程中应用最为广泛。

### 1.2 离子液体功能化磁性Fe_3_O_4_纳米材料的合成

ILs对Fe_3_O_4_ NPs的功能化修饰主要分为物理涂覆法和化学键合法两大类^［28-33］^。物理涂覆法依赖于非共价相互作用，操作简便但结合力较弱；化学键合法则基于共价键形成稳定连接，显著提高了材料的稳定性和耐久性。

#### 1.2.1 物理涂覆法

物理涂覆法是利用氢键、范德华力及疏水相互作用等非共价键作用力，将ILs固定于 Fe_3_O_4_ NPs或功能化的Fe_3_O_4_ NPs（图1a）。该方法主要通过超声、机械搅拌或溶剂挥发等方式制备IL-Fe_3_O_4_ NPs复合材料^［22，34，35］^。尽管存在稳定性欠佳、ILs易脱落分离的问题，但其操作简便且快速，是功能化Fe_3_O_4_ NPs最常用的方法之一。Bakheet等^［36］^将1-辛基-3-甲基咪唑六氟磷酸盐与磁性二氧化硅材料混合，经搅拌、二氯甲烷洗涤及60 °C真空干燥并研磨后，成功制得表面呈海绵状粗糙结构的磁性复合材料，并将其用于分离食品样品中的藏红T。

**图1:**
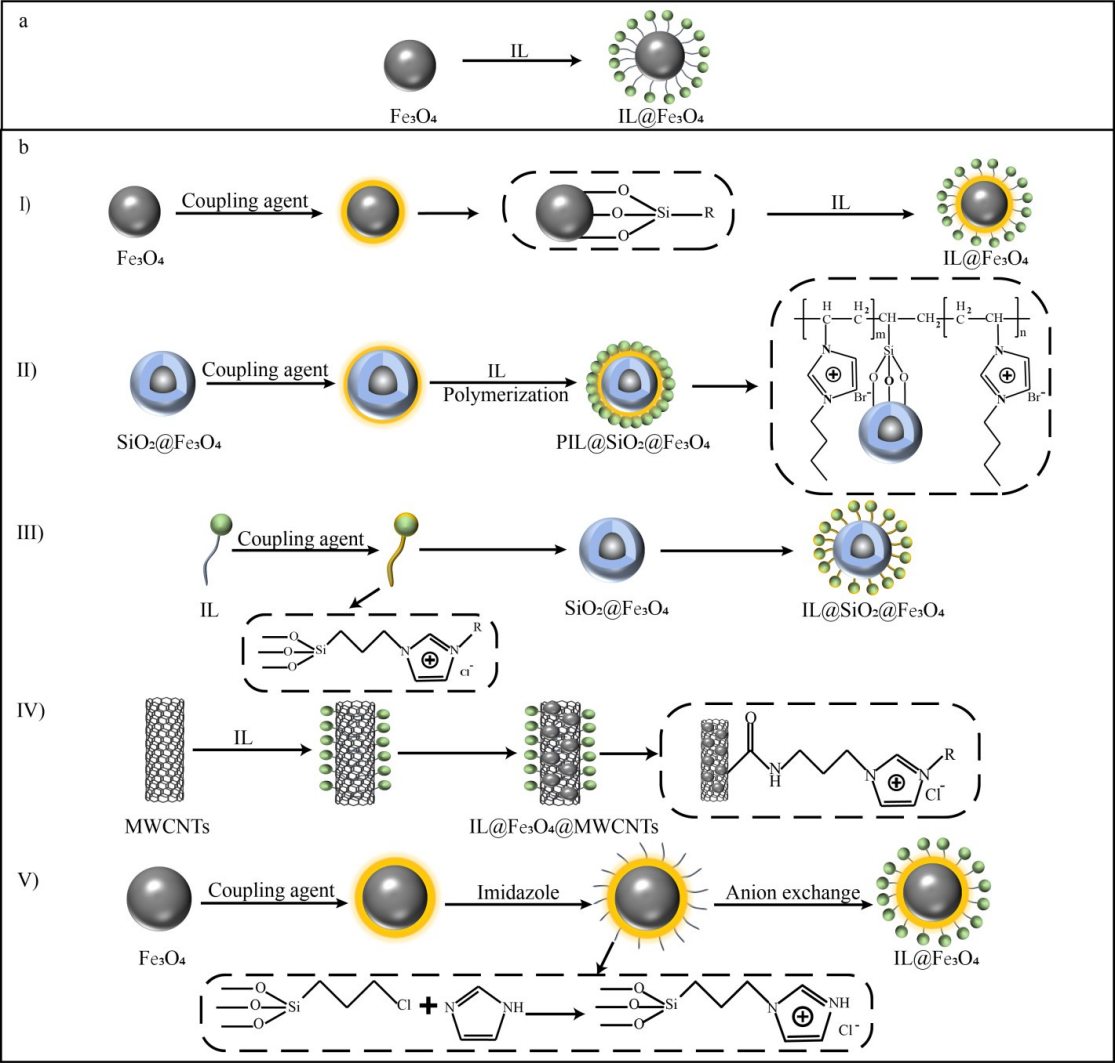
IL-Fe_3_O_4_ NPs的合成过程示意图

#### 1.2.2 化学键合法

与物理涂覆法相比，化学键合法制备的IL-Fe_3_O_4_ NPs更稳定、更易重现，并且延长了材料的使用寿命，其键合方式包括直接法和间接法^［14］^。

直接法是将功能化的ILs以共价键的形式与Fe_3_O_4_ NPs表面的官能团相结合的方法，其主要包括偶联剂法和表面接枝聚合法两种。第一种方法利用偶联剂（硅烷偶联剂或异氰酸酯偶联剂），在Fe_3_O_4_ NPs表面引入氨基（-NH_2_）、巯基（-SH）等活性基团，再与ILs通过化学反应发生共价结合^［19，37，38］^（图1bⅠ ）；第二种方法则是以2，2′-偶氮二异丁腈或过硫酸铵为引发剂，通过自由基共聚反应将ILs单体聚合在硅烷化的Fe_3_O_4_ NPs表面，形成聚合离子液体包覆结构^［39-41］^（图1b Ⅱ）。Wang等^［42］^通过第一种方法以1，6-二异氰酸己烷作为偶联剂将1-乙基-3-甲基咪唑L-脯氨酸盐直接键合于Fe_3_O_4_ NPs表面，成功制备出具有约20 nm核壳结构的磁性纳米材料。该材料对血液中血红蛋白的吸附结合容量高达1.58 mg/mg，可在30 s内实现与样品溶液的快速磁性分离。李丹娜等^［43］^采用第二种方法以2，2′-偶氮-二异丁腈为引发剂，在甲醇中通过自由基共聚反应将1-乙烯基-3-（12-溴代十二烷基）咪唑溴盐固定在磁性二氧化硅纳米材料表面，制备了用于富集油样中呕吐毒素的新型吸附剂材料。

间接法主要包括3种方法：第一种方法，先将ILs硅烷化，然后将硅烷化的ILs共价键合到Fe_3_O_4_ NPs的表面^［44，45］^（图1b Ⅲ）；第二种方法，首先将ILs引入到纳米材料中制备离子液体基纳米材料，之后将再与Fe_3_O_4_ NPs结合^［46］^（图1b Ⅳ），该方法可避免材料的高温氧化，但纳米材料的空间位阻会限制ILs的键合数量；第三种方法，硅烷材料对Fe_3_O_4_ NPs表面进行修饰，随后通过化学键将咪唑环接枝到Fe_3_O_4_ NPs表面，进而形成类似于ILs的结构，最后通过阴离子交换获得目标IL-Fe_3_O_4_ NPs（图1b V），该方法在IL-Fe_3_O_4_ NPs制备过程中形成的ILs结构可以避免单独制备ILs所需的纯化过程^［11，47］^。Liu等^［45］^采用第一种方法以1-十八烷基咪唑鎓氯盐为功能单体，通过硅烷化反应将其键合于磁性二氧化硅表面，成功合成了具有核壳结构的磁性纳米吸附剂，并将其应用于蜂蜜中黄酮类化合物和肉桂中肉桂酸的富集。Chen等^［46］^采用第二种方法将1-（3-氨基丙基）咪唑鎓氯盐通过酰胺键固定于羧基化多壁碳纳米管表面，随后与Fe_3_O_4_ NPs复合制备功能化磁性碳纳米管。在提取环境水样中的三唑类杀菌剂时，该材料表现出良好磁性，在外加磁场作用下可在20 s内实现与样品溶液的快速分离。Yamini等^［22］^以第三种方法先利用3-氯丙基三甲氧基硅烷对磁性二氧化硅进行氯丙基衍生化，随后引入*N*-甲基咪唑形成甲基咪唑鎓氯盐修饰的Fe_3_O_4_ NPs，最后将六氟磷酸根置换为氯离子，成功制备了用于富集制革废水样品中镉离子的功能化磁性吸附剂。

## 2 离子液体功能化磁性Fe_3_O_4_纳米材料的分类

在IL-Fe_3_O_4_ NPs中，ILs直接修饰Fe_3_O_4_ NPs存在比表面积低、吸附位点不足的问题。通过在Fe_3_O_4_ NPs表面包覆二氧化硅、碳基材料或金属有机骨架材料等载体进行复合改性，可显著提升材料性能（图2）。在此基础上，进一步引入ILs使其兼具ILs和复合载体的双重特性，还提高了传质性能，赋予材料更优异的吸附解吸能力^［12］^。从阳离子结构的角度，ILs可分为咪唑基、吡啶基、季氨基、胍基及1，4-二氮杂双环［2.2.2］辛烷基等；而根据阴离子的结构可分为氯离子、溴离子、碘离子、四氟硼酸根、六氟磷酸根、双（三氟甲磺酰）亚胺、氨基酸等^［19，48-50］^（图3）。经过ILs与多种材料的多级化修饰，Fe_3_O_4_ NPs的性能得到显著优化（具体优缺点见表1），使其对复杂样品中的痕量组分富集效率大幅提升。

**图2:**
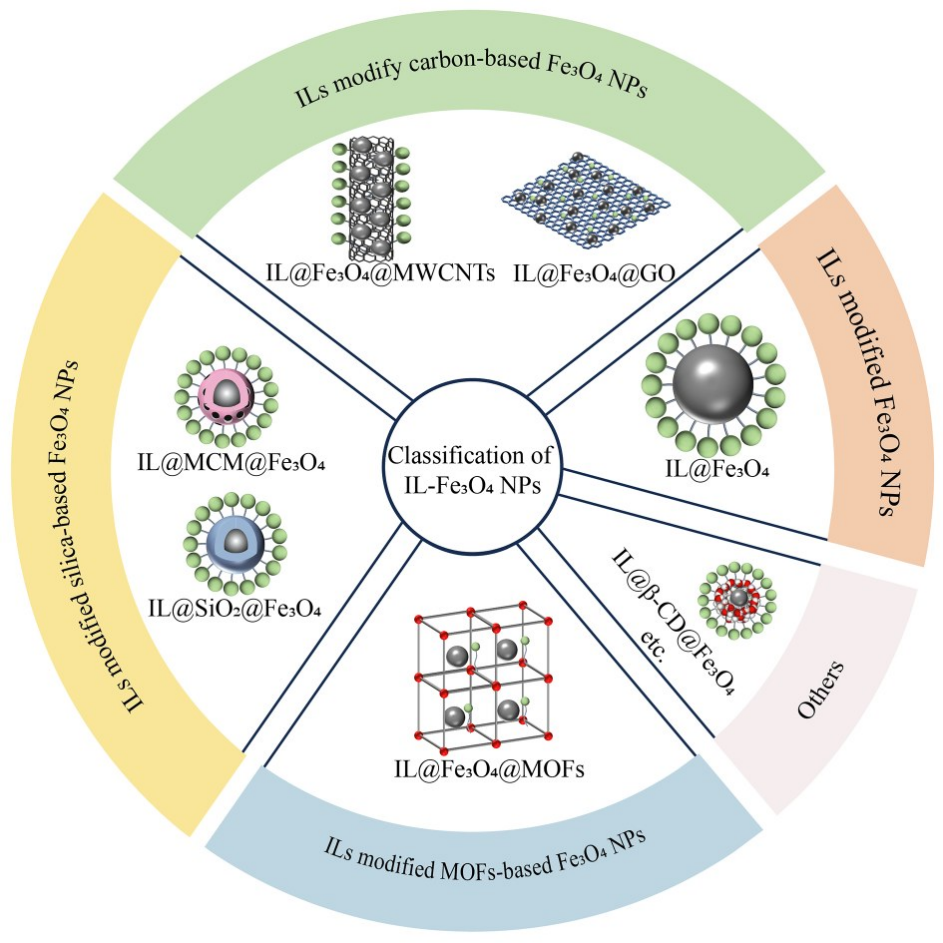
IL-Fe_3_O_4_ NPs的分类示意图

**图3:**
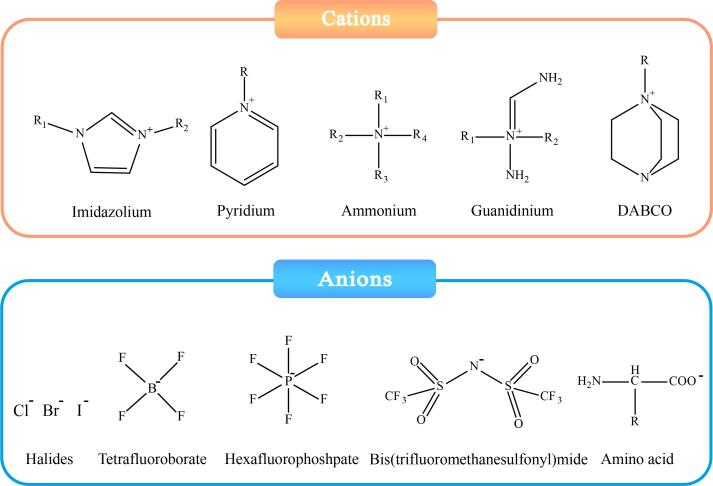
ILs的分类示意图

**表1:** IL-Fe_3_O_4_ NPs的优缺点

Materials	Advantages	Disadvantages	Ref.
ILs modified Fe_3_O_4_ NPs	simple synthesis， low cost， high magnetic response， easy to separate and recycle	low specific surface area， small adsorption capacity	［^13^］
ILs modified silica-based Fe_3_O_4_ NPs	magnetic responsiveness， easy to separate and recycle， good biocompatibility， SiO_2_ shell improves chemical stability and corrosion resistance， mesoporous structure improves load and mass transfer efficiency， easy surface functionalization， high compatibility with various ILs	SiO_2_ shell may mask magnetic core， reducing magnetic responsiveness	［^37^，^39^］
ILs modified carbon-based Fe_3_O_4_ NPs	magnetic responsiveness， easy to separate and recycle， high specific surface area， abundant *π*-electron system， numerous active sites， excellent chemical stability， high temperature and acid/alkali resistant	high cost， carbon coating induced magnetic attenuation	［^51^，^52^］
ILs modified MOFs-based Fe_3_O_4_ NPs	strong magnetic responsiveness， facile separation， efficient recyclability， enhanced adsorption capacity， high porosity， tunable pore size， abundant unsaturated metal sites， effective coordination capability	high cost， complex synthesis process	［^53^，^54^］

### 2.1 离子液体直接修饰Fe_3_O_4_纳米材料

Fe_3_O_4_ NPs易在溶液中团聚，同时还会受到酸碱腐蚀或空气氧化^［55］^，可用咪唑基或1，4-二氮杂双环［2.2.2］辛烷基等ILs进行修饰^［19，28］^，也可引入含有氨基酸^［42］^、硫醇^［56］^等活性基团的功能化ILs进行有效改善。该类材料的萃取机理主要是基于ILs功能基团与目标物间的多重相互作用（如静电作用、配位键合、疏水效应）以及Fe_3_O_4_ NPs的磁响应特性，通过咪唑环或特定官能团的选择性结合，实现对蛋白质^［28，42］^、金属离子^［19，56］^等物质的高效吸附。Kamran等^［28］^将1-丁基-3-甲基咪唑溴盐、1-己基-3-甲基咪唑溴盐及1-辛基-3-甲基咪唑溴盐分别对Fe_3_O_4_ NPs进行了改性，并将其作为富集环境水样中溶菌酶的磁性吸附剂。

### 2.2 离子液体修饰二氧化硅基Fe_3_O_4_纳米材料

二氧化硅具有高稳定性、可修饰性、可控的尺寸及孔隙率等优点，能有效防止Fe_3_O_4_ NPs在液体中的团聚，同时SiO_2_表面丰富的硅羟基为ILs的修饰提供了活性位点^［57］^。SiO_2_主要分为无孔型和介孔型两类，其中介孔SiO_2_因其更大的比表面积和丰富的活性位点有效克服了传统无孔SiO_2_结合位点不足和提取效率低的问题，受到了很多学者的关注^［58］^。Fe_3_O_4_ NPs经SiO_2_包覆后，常与咪唑基^［59］^、吡啶基^［30］^、季铵基^［44］^、胍基^［60］^及1，4-二氮杂双环［2.2.2］^［37］^辛烷基等ILs结合，同时也可在ILs中引入磷酸^［16］^、磺酸^［17］^及氨基酸^［24］^等基团进行功能化改性。此材料萃取的机理有如下可能：Fe_3_O_4_ NPs赋予该材料快速分离能力，SiO_2_壳层提供高比表面积、吸附容量和可调节的孔径，二者与ILs协同发挥静电相互作用^［16，22，36］^（如磺酸基、咪唑阳离子与带电分析物间的静电吸引或排斥）、*π*-*π*堆积作用^［31，33，43］^（ILs中的芳香环与目标物芳香结构间的相互作用）、疏水作用^［29，45，47］^（ILs烷基链或疏水基团与非极性分子的亲和）、氢键作用^［37，39］^（羟基等极性基团与目标物的键合）、配位作用^［16，32］^（如Ti^4+^与磷酸基团的螯合），可高效选择性吸附金属离子^［22］^、染料^［30］^、农药^［24］^等目标化合物。Zhao等^［39］^通过表面引发原子转移自由基聚合将1-乙烯基咪唑接枝到磁性介孔SiO_2_纳米材料表面，制备出一种对苹果中除虫菊酯类农药表现出高吸附容量的磁性吸附剂。该吸附剂的介孔结构提供高吸附容量，IL功能基团与农药间的*π*-*π*堆积、氢键相互作用增强选择性，磁性内核可实现快速分离，1 min内即可达到吸附平衡，简化了萃取过程。

### 2.3 离子液体修饰碳基Fe_3_O_4_纳米材料

碳基材料是一类由碳元素构成的、涵盖零维到三维的纳米材料^［61］^。当Fe_3_O_4_ NPs经碳基材料修饰后，不仅融合了碳材料和磁性材料的优势特性，还降低了Fe_3_O_4_ NPs的团聚倾向，同时借助碳基材料的*π*-*π*堆积、疏水作用及表面功能基团显著提升了材料的选择性^［62］^。碳基材料修饰的Fe_3_O_4_ NPs常与咪唑基^［46，51，52］^、二氮卓双环［2.2.2］辛烷基^［18］^等ILs结合，也引入含羟基^［63］^、氨基酸^［64］^等功能基团的ILs或具有三维结构的ILs^［65］^，以优化其性能。在该类复合材料中，Fe_3_O_4_ NPs赋予材料超顺磁性以实现快速磁分离，碳基载体提供高比表面积和丰富的*π*电子体系，可通过*π*-*π*堆积、疏水作用及范德华力高效吸附含芳香环或疏水基团的目标物（如农药、酚类化合物、抗生素等）^［66-68］^。此外，ILs的功能化进一步增强了吸附选择性和容量：静电作用强化带电分子结合^［69，70］^，配位作用螯合金属离子^［34，71，72］^，氢键键合极性化合物^［73-75］^，而疏水性ILs可形成半胶束结构富集非极性物质^［51，63，67］^。Zhang等^［75］^通过将1-氨丙基-3-甲基咪唑溴化盐修饰的磁性碳纳米管作为吸附剂，利用静电作用、*π*-*π*堆积及氢键作用，实现了牛奶和猪肉中痕量氟喹诺酮类药物的高效萃取。

### 2.4 离子液体修饰金属有机骨架基Fe_3_O_4_纳米材料

在金属有机骨架材料的表面或孔隙中引入Fe_3_O_4_ NPs，可保留其固有结构特性并赋予材料磁性，同时增强结构稳定性及可回收性。而咪唑基离子液体的进一步功能化修饰，可显著提升复合材料的化学稳定性和分散性。该类材料萃取的机理有如下可能：金属有机骨架通过周期性孔道结构和丰富的配位不饱和金属位点提供吸附位点^［76］^；Fe_3_O_4_ NPs不仅赋予材料快速磁分离能力，其表面羧基化修饰可通过氢键作用和静电相互作用捕获极性污染物^［23］^；ILs的修饰增强了材料的吸附能力，其阴阳离子及疏水烷基链通过离子交换和静电作用、疏水效应和*π*-*π*堆积作用高效吸附非极性及芳环类化合物^［77，53］^，同时含有羟基、羧基等极性官能团的ILs与目标物易形成氢键网络或Lewis酸碱配位^［54］^。陈坤等^［23］^基于水热合成法制备了1-己基-3-甲基咪唑溴化盐功能化磁性MIL-101新型材料，并用于牛奶中黄曲霉毒素的富集。在萃取过程中，该材料利用其高比表面积及功能化基团与目标物存在的氢键作用、*π*-*π*堆积作用、静电相互作用共同实现了对黄曲霉毒素的高效吸附。

### 2.5 离子液体修饰其他大分子、小分子基Fe_3_O_4_纳米材料

除了以上材料，壳聚糖、聚苯胺、聚乙二醇、环糊精及纤维素等功能材料同样可用于包覆Fe_3_O_4_ NPs。壳聚糖是一种天然可再生资源，具有良好的生物降解性和生物相容性，包覆Fe_3_O_4_ NPs后，可结合氧化石墨烯或金属有机骨架材料制备复合材料，而胍基ILs及咪唑基ILs的功能化进一步增强了磁性材料的机械性能^［49，78-80］^。聚苯胺因具有良好的环境稳定性、易于合成且相对较低的成本，可通过与双阳IL形成氢键作用，增强磁性材料的热稳定性^［81，82］^。聚乙二醇凭借生物相容性和柔性网络结构，既能防止Fe_3_O_4_ NPs聚集，又可通过咪唑基或双阴离子氨基酸型离子液体修饰形成特定电荷分布^［83，84］^。此外*β*-环糊精、纤维素及杯芳烃制备的IL-Fe_3_O_4_ NPs在绿色样品前处理领域也具有很大的前景^［14，85-88］^。针对不同分析物的前处理需求，IL-Fe_3_O_4_ NPs可实现多元化设计，应用前景广阔。

## 3 离子液体功能化磁性Fe_3_O_4_纳米材料的萃取模式

近年来，IL-Fe_3_O_4_ NPs在样品前处理领域展现出显著优势，主要应用于MSPE、IT-SPME和PT-SPE等前处理方法。其中，MSPE是IL-Fe_3_O_4_ NPs的主萃取模式，Fe_3_O_4_ NPs的快速磁分离特性大幅提升了萃取效率；IT-SPME是将IL-Fe_3_O_4_ NPs固定于毛细管内壁，实现了与色谱系统的在线联用；而PT-SPE则利用IL-Fe_3_O_4_ NPs填充的移液吸头，简化微量样品的处理流程，尤其适用于痕量目标物的快速捕获。

### 3.1 磁性固相萃取

磁性固相萃取是将磁性吸附剂分散于样品溶液中对目标物进行吸附、利用外加磁场实现目标物与基质的快速分离、经过洗脱后用于检测的萃取方法^［6］^（图4a）。IL-Fe_3_O_4_ NPs作为MSPE的吸附剂，兼具高效选择性吸附、快速磁分离、操作便捷、功能可调及环境友好等优势。此外，该技术可与超临界萃取^［44］^、分散液液微萃取^［30］^等技术联用，或整合至QuEChERS体系中^［41］^。Badragheh等^［31］^将由自由基共聚反应制备的1-乙烯基-3-辛基咪唑六氟磷酸盐功能化磁性SiO_2_作为MSPE的吸附剂，并结合高效液相色谱（HPLC）同时测定人血浆中微量抗糖尿病药物，在最优条件下，恩格列净、二甲双胍和卡格列净的检出限分别为1.3、6.0和0.80 ng/mL，回收率为86.5%~92.5%。随着MSPE技术的发展，Gao等^［89］^将1-烷基-3-甲基咪唑盐、Fe_3_O_4_ NPs与碳酸钠混合制成磁性泡腾片，利用泡腾反应扩大与有机磷农药的接触面积，并结合HPLC检测水样中的有机磷农药，方法优化后，对甲胺磷、对硫磷及辛硫磷农药的检出限为0.14~0.22 mg/L，回收率为81.4%~97.6%。

**图4:**
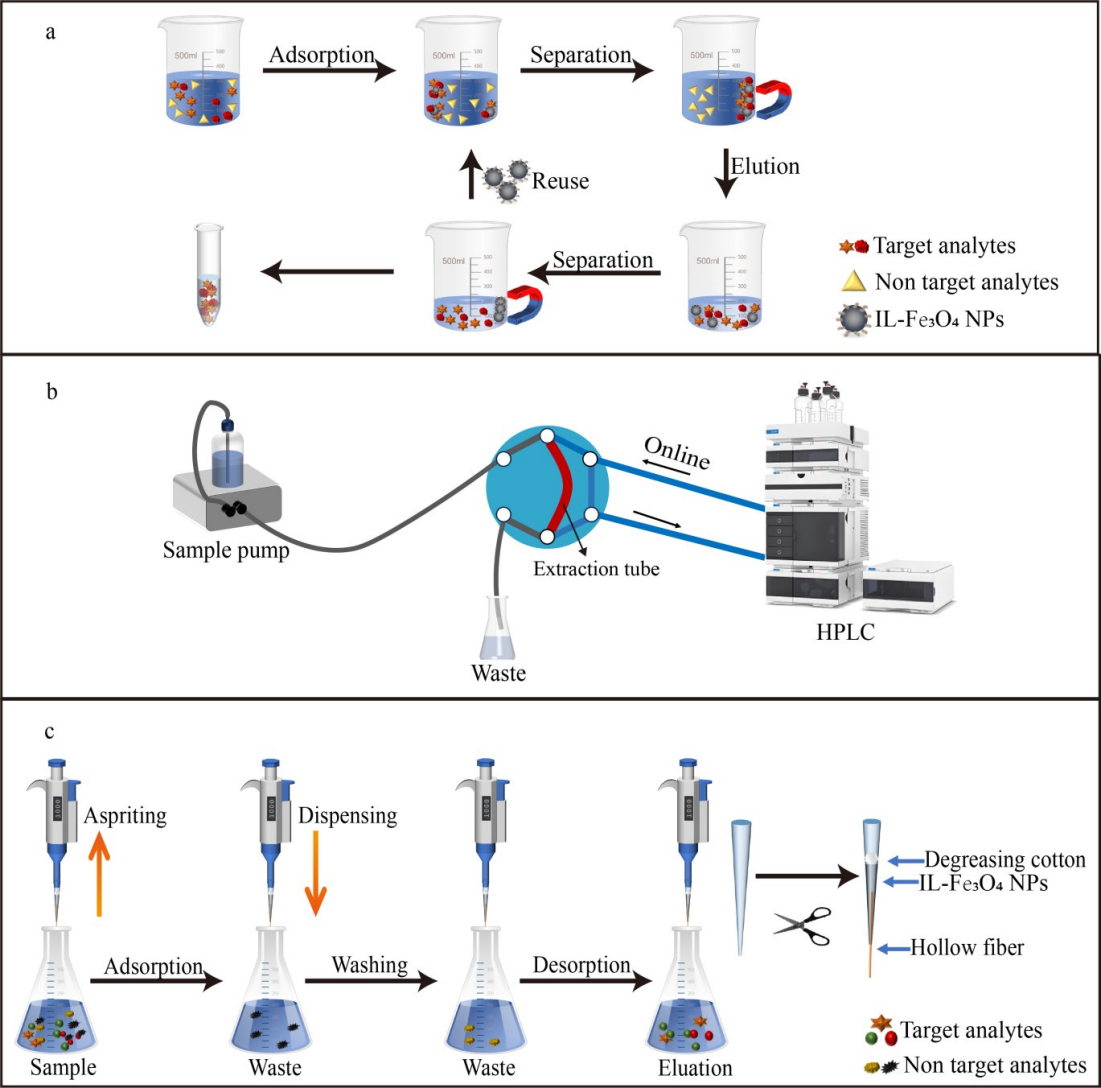
IL-Fe_3_O_4_ NPs的萃取模式示意图

### 3.2 管内固相微萃取

IT-SPME是一种基于毛细管内壁固定相吸附的微型化萃取技术，其主要通过转动六通阀切换流路来实现分析物的萃取与洗脱^［90］^（图4b）。该技术可与检测技术实现在线联用，形成从样品前处理到分析检测的一体化流程，具有操作简便、快速、高效、无需溶剂、样品用量少且易于实现自动化等优点。IL-Fe_3_O_4_ NPs作为IT-SPME的萃取材料，避免了传统涂层易脱落的问题，使得萃取时间缩短，且可实现萃取管的重复使用，显著提升了检测灵敏度的稳定性和方法的可持续性。Mei等^［20］^使用新型离子液体1-烯丙基-3-甲基咪唑双［（三氟甲基）磺酰基］酰亚胺作为功能单体制备了基于聚合离子液体的磁性整体柱，作为IT-SPME的萃取介质，并结合HPLC在线分析了环境水样中5种有机紫外线过滤剂，通过方法优化，有机紫外线过滤剂的检出限为0.040~0.26 g/L，回收率为70.5%~119%。

### 3.3 移液吸头固相萃取

PT-SPE是一种微型、快速、便携的固相萃取技术，其主要通过填充在移液吸头中吸附剂的反复吸附和解吸过程而完成分析物的提取（图4c）。该技术不仅保留了传统固相萃取的优点，还进一步减少了溶液和吸附剂的用量，具有操作灵活、简便、快速，提取效率高以及通用性强的特点^［91］^。当IL-Fe_3_O_4_ NPs为吸附剂时，其强吸附能力和优异的稳定性显著提升了PT-SPE的重现性，分析物仅通过少量次数的吸排循环即可实现充分富集，且洗脱液无需真空浓缩即可直接进样分析。Zhang等^［21］^将三维离子液体-铁氧体功能化氧化石墨烯纳米复合材料作为PT-SPE的吸附剂，对人体血液样本中的多环芳烃进行了分析。在优化条件下，多环芳烃的检出限为0.007 0~0.013 µg/L，回收率为85.0%~115%。该方法不仅实现吸附剂重复使用10次以上仍保持稳定性能，同时将样品消耗量降低至百微升级，充分体现了IL-Fe_3_O_4_ NPs基PT-SPE技术在痕量分析中的高效性和可持续性优势。

## 4 检测方法

在分析化学领域，对于复杂基质中目标化合物的精准检测与分析一直是研究的重点与核心。随着科技的飞速发展，各种先进分析技术不断涌现，各种色谱、光谱、质谱及联用技术为痕量目标化合物的检测和多组分的联合分析提供了有力的工具和手段。

### 4.1 在线模式

样品前处理是分析检测过程中耗时最多、最容易引起误差的步骤，因此样品前处理技术与色谱、光谱及各种联用技术的在线联用研究一直是分析化学的研究重点。在线技术可降低分析成本，节省时间和人力，同时减少手工操作中的样品差异，提高灵敏度、准确度和重现性^［92］^。基于IL-Fe_3_O_4_ NPs的IT-SPME技术可与HPLC实现环境样品中有机物的在线分析^［20］^。在Mei等^［20］^的研究中，以1-烯丙基-3-甲基咪唑双三氟甲磺酰亚胺盐为功能单体，乙二醇二甲基丙烯酸酯为交联剂，通过原位聚合将经硅烷化修饰的Fe_3_O_4_ NPs，形成具有均匀孔结构和超顺磁性的有机紫外滤光剂萃取介质。该介质集成于六通阀在线系统，在0.12 mL/min流速下完成样品吸附后，切换阀门以20 µL/min注入甲醇进行梯度洗脱解吸，结合梯度洗脱实现20 min内全自动富集-检测。

### 4.2 离线模式

离线检测方法仍是最常用的分析方法。因IL-Fe_3_O_4_ NPs对金属离子的优异溶解能力，在金属离子富集中展现出突出优势。Khodadadi等^［32］^使用1-（2，3-二羟基丙基）-1，4-二氮杂双环［2.2.2］辛烷氯化物改性的Fe_3_O_4_ NPs进行MSPE，并结合火焰原子吸收分光光度法（FASS）测定牛奶样品中痕量的Pb（Ⅱ）和Cd（Ⅱ）。在最佳条件下，Pb（Ⅱ）和Cd（Ⅱ）的检出限为0.070~0.090 µg/L，回收率为91.6%~100.8%，且吸附剂可重复使用5次以上，兼具经济性与环保性，充分展现了原子吸收分光光度法技术与新型纳米材料前处理联用在实际检测中的应用潜力。在有机污染物检测方面，Shahriman等^［81］^采用双（1-苄基-3-咪唑鎓）对二甲苯双（三氟甲磺酰亚胺）涂覆聚苯胺改性的Fe_3_O_4_ NPs制备了磁性复合材料，将其作为MSPE的吸附剂，并结合气相色谱-质谱法（GC-MS）检测环境样品中的多环芳烃。该方法中多环芳烃的检出限低至0.001 0~0.21 μg/L，同时实现了80.2%~112%的萃取回收率。

色谱技术以其能够对各类样品进行高效分离分析而被广泛应用。然而，随着各类新型复杂样品体系的不断涌现，作为色谱分析前的重要组成部分，样品前处理对于实现高效色谱分离分析至关重要。IL-Fe_3_O_4_ NPs在样品前处理中展现出良好性能，其功能化设计适配不同检测技术并针对特定分析物：IL@Fe_3_O_4_目前已与UV-Vis、FASS或FASS-MS等光谱技术离线联用，可用于血红蛋白、溶菌酶等生物大分子及Cd（Ⅱ）、Cr（Ⅱ）、Pb（Ⅱ）等金属离子的检测^［42，56］^；IL@SiO_2_@Fe_3_O_4_则常与HPLC、GC、UV-Vis、电感耦合等离子原子发射光谱法（ICP-AES）等多种技术联用，实现对染料、天然产物等化合物的富集^［22，30，45，69］^；而IL@Fe_3_O_4_@carbon可与HPLC、GC-MS、UV-Vis等技术离线联用，有效分析极性与非极性有机污染物、重金属离子等物质^［72-75］^；IL@Fe_3_O_4_@MOFs可与HPLC技术结合用于检测农药^［77］^。此外，在ILs修饰其他大分子、小分子基Fe_3_O_4_纳米材料中，IL@Fe_3_O_4_@*β*-CD应用最广，能高效富集多种分析物并与多种检测技术（如HPLC、UPLC-MS/MS及UV-Vis等）联用，可用于分离手性化合物^［14，85，86］^。值得注意的是，该类材料已被拓展应用于色谱固定相，Yang等^［93］^分别将*β*-环糊精修饰的Fe_3_O_4_ NPs和IL修饰的磁性Fe_3_O_4_ NPs包被在毛细管内壁上作为毛细管电色谱的固定相，并用于分离6种丹磺酰化氨基酸的对映体。经参数优化后，两种毛细管柱电渗流迁移时间的相对标准偏差（RSD）分别为2.56%和2.28%，均表现出优异的重复性、耐用性和手性识别能力。

## 5 离子液体功能化磁性Fe_3_O_4_纳米材料在样品前处理中的应用

### 5.1 环境样品

随着工业化、经济发展和人口的快速增长，大量有毒有害的污染物被排放到环境中，对人类的健康和生态系统的安全造成严重影响。因此，对环境污染物进行准确的分析对环境检测至关重要。IL-Fe_3_O_4_ NPs因对目标分析物具有较高的选择性和灵敏度，被广泛应用于环境分析领域。如表2所示，IL-Fe_3_O_4_ NPs在环境样品检测中展现出卓越的污染物富集能力，常用于富集环境样品中的内分泌干扰物、重金属、染料等目标分析物，尤其对重金属的检测灵敏度最高。Cang等^［34］^采用氨基酸离子液体包覆的磁性氧化石墨烯为固相萃取的吸附剂，并结合ICP-AES对水样中Cr（Ⅲ）和Cr（Ⅵ）的形态进行分析。在最优条件下，金属离子的线检出限为0.19~0.41 μg/L，富集因子为25.7，且回收率为82.9%~112%，在环境修复领域显示出良好的应用前景。Qiao等^［60］^制备了一种新型氨基功能化六烷基氯胍离子液体，通过聚乙烯亚胺共价接枝到磁性材料表面合成用于MSPE的吸附剂，并结合HPLC-UV检测富集水中的多环芳烃。条件优化后，多环芳烃的检出限为0.050 ng/mL，加样回收率为80.0%~120%，与已有的研究结果相比，该方法在水中多环芳烃的提取和检测中具有更大的应用潜力。

**表2:** IL-Fe_3_O_4_ NPs在环境样品中的应用

Classification of analytes	Adsorbents	Samples	Analytes	Extraction technology	Detection methods	LOD	Ref.
Endocrine disruptors	Poly［AMIM］［NTf_2_］@SiO_2_@Fe_3_O_4_	lake water， river water	organic ultraviolet filters	IT-SPME	HPLC-DAD	0.040-0.26 μg/L	［^20^］
	［HMIM］［PF_6_］@Fe_3_O_4_@ZIF-8@MWCNTs	tap water	dichlorodiphenyltrichloroethane	MSPE	GC-MS/MS	0.0010-0.0070 µg/L	［^53^］
	［diPrNH_2_TMG］［Cl］@SiO_2_@Fe_3_O_4_	tap water， lake water	polycyclic aromatic hydrocarbons	MSPE	HPLC-UV	0.050 ng/mL	［^60^］
	［C_16_MIM］［Br］@Fe_3_O_4_@GO	water， tap water	chlorophenol	MHMSPE	HPLC-UV	0.10-0.13 µg/L	［^69^］
Metal ions	［C_6_MIM］［Ala］@SiO_2_@Fe_3_O_4_@GO	water	Cr（Ⅲ）， Cr（Ⅵ）	MSPE	ICP-OES	0.19-0.41 µg/L	［^34^］
	［MIM］［Ala］@SiO_2_@Fe_3_O_4_@GO	sewage	Cd（Ⅱ）	MSPE	ICP-OES	0.0010 ng/mL	［^64^］
	IL@*β*-CDCP@Fe_3_O_4_	city water， lake water	Mn（Ⅱ）， Mn（Ⅶ）	MSPE	ICP-OES	0.15-0.27 μg/L	［^85^］
Dye	［Hpy］［NTf_2_］@SiO_2_@Fe_3_O_4_	river water， tap water， drinking water， waste water	malachite green， crystal violet， methylene blue	DLLME-MSPE	HPLC-UV	0.030-0.050 μg/L	［^30^］
	IL@Fe_3_O_4_@MoS_2_@RGO	lake water， river water	methylene blue	MSPE	UV-Vis	6.4 µg/L	［^70^］
	DICAT@Fe_3_O_4_@PANI	industrial waste water， lake water	rhodamine	MSPE	UV-Vis	-	［^82^］
Pesticides	［BMIM］［Cl］@MCM@Fe_3_O_4_	sea water， spring water， agricultural water	organophosphates， carbamates， pyrethroids	MSPE	GC-μ-ECD	0.040-1.6 μg/kg	［^38^］
	［C_6_OHMIm］［Br］@Fe_3_O_4_@GO	field water， river water， surface water	triazine and urea herbicides	MSPE	HPLC-DAD	0.040-0.050 μg/L	［^63^］
Drugs	［BMIM］［Br］@Fe_3_O_4_@GO	sewage	sulfonamide antibiotic	MSPE	UPLC-MS/MS	0.75-1.5 ng/L	［^35^］
	IL-COOH@Fe_3_O_4_@UiO-67	river water	fluoroquinolone antibiotic	MSPE	HPLC-DAD	0.010-0.020 μg/L	［^54^］
Biotoxins	［BMIM］［Br］@Fe_3_O_4_@GO	water	microcystins	MSPE	UPLC-MS/MS	0.27-0.41 ng/L	［^52^］

Poly（［AMIM］［NTf_2_］）： polymerized 1-allyl-3-methylimidazolium bis（trifluoromethylsulfonyl）imide； ［HMIM］［PF_6_］： 1-hexyl-3-methylimidazolium hexafluorophosphate； ［diPrNH_2_TMG］［Cl］： 1，3-di（3-aminopropyl）tetramethylguanidinium chloride； ［C_16_MIM］［Br］： 1-hexadecyl-3-methylimidazolium bromide； ［C_6_MIM］［Ala］： 1-hexyl-3-methylimidazolium alaninate； ［MIM］［Ala］： 1-methylimidazolium alaninate； *β*-CDCP： *β*-cyclodextrin crosslinked polymer； ［Hpy］［NTf_2_］： *n*-hexylpyridinium bis（trifluoromethylsulfonyl）imide； RGO： reduced graphene oxide； DICAT： dicationic ionic liquid； PANI： polyaniline； ［BMIM］［Cl］： 1-butyl-3-methylimidazolium chloride； ［C_6_OHMIm］［Br］： 1-（3-hydroxypropyl）-3-methylimidazolium bromide； ［BMIM］［Br］： 1-butyl-3-methylimidazolium bromide； UiO-67： Universitetet i Oslo-67； MHMSPE： magnetic hydrophobic meticulous solid-phase extraction； DLLME-MSPE： dispersive liquid-liquid microextraction combined with magnetic solid-phase extraction.

### 5.2 食品样品

IL-Fe_3_O_4_ NPs在食品中痕量或超痕量目标分析物的萃取与富集方面有显著的作用，具有分离速度快且吸附位点丰富等优点。如表3所示，该材料最常用于富集食品样品中的农药、兽药、添加剂，对金属离子、生物毒素、内分泌干扰物及天然产物等目标分析物也具有良好的吸附效果。Chen等^［24］^以功能化氨基酸离子液体为单体、二氧化硅基材料多面体低聚倍半硅氧烷（polyhedral oligomeric silsesquioxane，POSS）为交联剂，制备了新型特异性磁性纳米粒子作为磁性分散固相萃取的吸附剂，并结合HPLC-MS检测果汁和人血清中的痕量苯并二氮杂卓类化合物。结果表明，该方法检出限为0.060~0.150 μg/L，回收率为81.9%~102%，为其他食品污染物的绿色快速检测方法提供了思路。Sahebi等^［78］^采用功能咪唑离子液体修饰磁性壳聚糖纳米颗粒为吸附剂，结合UHPLC-MS/MS浓缩和测定了牛奶样品中22种抗生素及其代谢物，各种抗生素及其代谢物的检出限为0.040~0.19 μg/kg，回收率为85.9%~108%。

**表3:** Fe_3_O_4_ NPs在食品样品中的应用

Classification of analytes	Adsorbents	Samples	Analytes	Extraction technology	Detection methods	LOD	Ref.
Pesticides	Poly（［VPImi-SO_3_H］［Cl］）@SiO_2_@Fe_3_O_4_	vegetable	diquat	MSPE	HPLC-UV	0.090 μg/g	［^17^］
	［C_8_OBIM］［Gly］@SiO_2_@Fe_3_O_4_	fruit juice serum	benzimidazoles	MSPE	HPLC-MS/MS	0.060-0.150 μg/L	［^24^］
	PIL@mSiO_2_@nSiO_2_@Fe_3_O_4_	apple	pyrethroids	MSPE	GC-MS	0.24-2.0 ng/g	［^39^］
	［BMIM］［PF_6_］@HP-*β*-CD@Fe_3_O_4_	honey	carbofuranin	MSPE	HPLC-MS/MS	0.40 μg/kg	［^86^］
Veterinary drugs	［DABCO-C_3_OH］［Cl］@SiO_2_@Fe_3_O_4_	milk	penicillin	D-micro-SPE	UPLC-MS/MS	0.030-0.20 µg/kg	［^37^］
	［APMIM］［Br］@Fe_3_O_4_@MWCNTs	milk， pork	fluoroquinolone antibiotic	MSPE	HPLC-UV	0.33-0.78 ng/mL	［^75^］
	IL@Fe_3_O_4_@CS	milk	antibiotics and their metabolites	MSPE	UPLC-MS/MS	0.040-0.19 μg/kg	［^78^］
Additive	［OMIM］［PF_6_］@SiO_2_@Fe_3_O_4_	tomato sauces	safranine T	MSPE	UV-Vis	0.37 ng/mL	［^36^］
	Poly（［VOIM］［Br］）@SiO_2_@Fe_3_O_4_@G	vegetable	preservatives	QuEChERS	GC-MS/MS	0.020-0.42 μg/L	［^41^］
	［BMIM］［Trp］@SiO_2_@Fe_3_O_4_@GO	pepper， water	sudanⅠ-Ⅳ	MSPE	HPLC-UV	0.010-0.50 µg/mL	［^73^］
Metal ions	［DABCO-PDO］［Cl］@SiO_2_@Fe_3_O_4_	milk	Pb（Ⅱ）， Cd（Ⅱ）	MSPE	FAAS	0.070-0.090 µg/L	［^32^］
	［HMIM］［PF_6_］@SiO_2_@Fe_3_O_4_@GO	shellfish	Pb（Ⅱ）， Cu（Ⅱ）， Cr（Ⅱ）	d-MSPE	ICP-MS	2.4-3.8 ng/L	［^67^］
Biological toxins	［HMIM］［Br］@Fe_3_O_4_-COOH@MIL-101	milk	aflatoxin	MSPE	HPLC-FLD	0.030-0.15 μg/L	［^23^］
	Poly（［VDIm］［Br］）@SiO_2_@Fe_3_O_4_	vegetable oil	vomitoxin	MSPE	UHPLC-UV	3.3 μg/kg	［^43^］
Endocrine disruptors	［C_4_MIM］［PF_6_］@HP-*β*-CD@Fe_3_O_4_	drink	bisphenol A	MSPE	HPLC-FLD	0.40 μg/L	［^14^］
	3D-［APMIM］［Br］@Fe_3_O_4_@GO	vegetable oil	polycyclic aromatic hydrocarbons	MSPE	GC-MS	0.10-0.60 μg/kg	［^65^］
Secondary metabolites	［VOIM］［Br］@SiO_2_@Fe_3_O_4_@CS@GO	coffee， milk tea， hot pot seasoning	alkaloids	MSPE	UPLC-MS/MS	-	［^79^］
	Poly（CalixIL）@SiO_2_@Fe_3_O_4_	fruit juice， green tea	flavonoids	MSPE	HPLC-DAD	0.15-0.75 ng/mL	［^88^］

Poly（［VPImi-SO_3_H］［Cl］）： polymerized ionic liquid based on 1-vinyl-3-（3-sulfopropyl）imidazolium chloride； ［C_8_OBIM］［Gly］： 1-methyl-3-（octyloxy）imidazolium glycinate； PIL： poly ionic liquid； ［BMIM］［PF_6_］： 1-butyl-3-methylimidazolium hexafluorophosphate； HP-*β*-CD： hydroxypropyl-*β*-cyclodextrin； ［DABCO-C_3_OH］［Cl］： 1-（3-hydroxypropyl）-1，4-diazabicyclo［2.2.2］octanium chloride； ［APMIM］［Br］： 1-（3-aminopropyl）-3-methylimidazolium bromide； CS： chitosan； ［OMIM］［PF_6_］： 1-octyl-3-methylimidazolium hexafluorophosphate； Poly（［VOIM］［Br］）： polymerized ionic liquid based on 1-vinyl-3-octylimidazolium bromide； ［BMIM］［Trp］： 1-butyl-3-methylimidazolium tryptophanate； ［DABCO-PDO］［Cl］： 1-（2，3-dihydroxypropyl）-1，4-diazabicyclo［2.2.2］octanium chloride； ［HMIM］［Br］： 1-hexyl-3-methylimidazolium bromide； MIL-101： Matériaux de l'Institut Lavoisier-101； Poly（［VDIm］［Br］）： polymerized ionic liquid based on 1-vinyl-3-decylimidazolium bromide； ［C_4_MIM］［PF_6_］： 1-butyl-3-methylimidazolium hexafluorophosphate； Poly（CalixIL）： polymerized calixarene-based ionic liquid； d-MSPE： dispersive magnetic solid-phase extraction； D-micro-SPE： dispersive micro-solid-phase extraction； QuEChERS： quick， easy， cheap， effective， rugged， and safe.

### 5.3 生物样品

生物样品基质复杂且目标分析物含量低，对样品前处理技术提出了更高要求。IL-Fe_3_O_4_ NPs因其优异的生物相容性、可调控的表面化学性质以及快速的磁响应特性，在生物样品前处理中展现出独特优势。如表4所示，IL-Fe_3_O_4_ NPs在生物样品检测中最适用于小分子药物和内分泌干扰物的分析，对生物大分子的检测灵敏度较低。Jiang等^［16］^将膦酸酯官能化离子液体固定在核壳结构的磁性介孔纳米材料的表面制备了纳米材料，将其作为MSPE的吸附剂，并结合基质辅助激光解吸飞行时间质谱捕获生物样品中痕量的磷酸肽。在最佳条件下，该材料可重复使用至少12次，具有优异的稳定性。Liu等^［49］^制备了一系列胍盐离子液体修饰的磁性壳聚糖/氧化石墨烯纳米复合材料作为DNA提取的吸附剂，并以微量紫外可见分光光度计评估提取效率。在最佳提取条件下，纳米复合材料的最大DNA提取容量达到（233.0±0.4） mg/g。

**表4:** IL-Fe_3_O_4_ NPs在生物样品中的应用

Classification of analytes	Adsorbents	Samples	Analytes	Extraction technology	Detection methods	LOD	Ref.
Biomolecule	［PFIL-Ti^4+^］@mSiO_2_@Fe_3_O_4_	protein solution， saliva	phosphopeptides	MSPE	MALDI-TOFMS	-	［^16^］
	Poly（［APr-VBIM］［Cl］）@Fe_3_O_4_@MWCNTs	porcine whole blood	Cu， Zn-superoxide dismutase	MSPE	UV-Vis	-	［^40^］
	HDI-［EMIM］［Lpro］@Fe_3_O_4_	blood	hemoglobin	MSPE	UV-Vis	-	［^42^］
	GIL@Fe_3_O_4_@GO	single-stranded DNA samples， salmon sperm DNA， sodium salt， etc.	DNA	MSPE	UV-Vis	-	［^49^］
	DAAAIL@Fe_3_O_4_@PEG	rude bovine， porcine pancreas	trypsin	MSPE	UV-Vis	3.1 μg/mL	［^83^］
Drugs	Poly（［VOIM］［PF_6_］）@Fe_3_O_4_@SiO_2_	plasma	empagliflozin， metformin and canagliflozin	MSPE	HPLC-UV	0.80-6.0 ng/mL	［^31^］
	IL@SiO_2_@Fe_3_O_4_	blood	tolmetin， indometacin， naproxen	SPE-MSPE	HPLC-UV	0.20-0.30 mg/kg	［^44^］
	［HMIM］［PF_6_］@Fe_3_O_4_@G	urine	tramadol	MHMDSPE	HPLC-UV	12 ng/mL	［^51^］
Endocrine disruptors	3D-IL@Fe_3_O_4_@GO	blood	polycyclic aromatic hydrocarbons	PT-SPE	GC-MS	0.0020-0.0040 µg/L	［^21^］

［PFIL-Ti^4+^］： phosphonate-functionalized ionic liquid with Ti⁴⁺ ions； Poly（［APr-VBIM］［Cl］）： poly（1-allyl-3-vinylimidazolium chloride）； PEG： polyethylene glycol； DAAAIL： double amino acid ionic liquid； HDI： 1，6-diisocyanatohexane； ［EMIM］［Lpro］： 1-ethyl-3-methylimidazolium l-prolinate； GIL： guanidinium ionic liquid； Poly（［VOIM］［PF_6_］）： polymerized 1-vinyl-3-octylimidazolium hexafluorophosphate.

## 6 总结与展望

IL-Fe_3_O_4_ NPs兼具Fe_3_O_4_ NPs独特的磁分离特性和ILs优良的吸附特性，具有良好的稳定性、选择性以及丰富的吸附位点，在样品前处理领域展现出了巨大的应用潜力。其制备方法主要包括物理涂覆法和化学键合法，其中，化学键合法是最常见的合成策略。而IL-Fe_3_O_4_ NPs的萃取模式主要有MSPE、IT-SPME和PT-SPE，其中MSPE是主要的萃取模式，大幅提高了对各类目标分析物的萃取效率。

基于IL-Fe_3_O_4_ NPs的样品前处理技术可与多种检测技术实现在线或离线联用，主要的色谱法包括HPLC，光谱法主要为UV-Vis、ICP-OES、FASS、原子荧光光谱法；联用技术主要包括LC-MS联用和GC-MS联用；其中HPLC可实现与前处理方法的在线联用。这些检测技术极大地提高了复杂样品分析中目标化合物的灵敏度、选择性和准确性，为各领域的研究和应用提供了强有力的支撑。

迄今为止，IL-Fe_3_O_4_ NPs已广泛应用于环境、食品及生物样品领域的前处理。然而，部分IL-Fe_3_O_4_ NPs的合成工艺仍依赖高温反应条件，存在能耗高、可控性不足等问题。为此，一锅法、光诱导及pH诱导等合成策略可能用于制备IL-Fe_3_O_4_ NPs，以降低反应温度并提升材料制备效率。随着功能化ILs及复合材料的持续发现，其发展方向可能聚焦于合成环境友好性ILs，并可探索低共熔溶剂、超分子溶剂等绿色溶剂对各种MNPs的功能化修饰，从而增强其生物相容性和选择性吸附性能。另外，基于IL-Fe_3_O_4_ NPs的样品前处理技术已与HPLC实现在线联用，未来有望进一步与更多的色谱、光谱及联用技术形成一体化分析体系。综上所述，IL-Fe_3_O_4_ NPs会在更多领域发挥重要作用，为样品前处理技术的发展带来新的突破，推动相关领域的科学研究和应用的发展。
